# 

**Published:** 1915-01-02

**Authors:** 


### The East Coast Hospitals and the Wounded.

No new convoys have been lately received at Beckett Hospital, Barnsley, and, generally speaking, it seems to be the impression that many available hospitals near the East Coast are at present being given a rest, and perhaps it is as well in case there should be any urgent demand for them. A new convalescent home has been opened within the borough, where some twelve beds are available. It is situated near Locke Park, which is a very pleasant and suitable district for exercising. The entire cost of the home is being defrayed by Mr. E. G. Lancaster, J.P. The care of the patients is under the superintendence of Miss Belamy, a former matron of Beckett Hospital, and the medical supervision has been kindly undertaken by Dr. Sadler, M.D. The Lunn Wood Hospital has a full contingent of wounded British soldiers, who are all progressing favourably.

### Buildings Contemplated.

Macclesfield.—It is proposed to extend the hospital at a cost of about £1,000.

Marple.—The Salford Town Council have agreed to apply to the Local Government Board for sanction to borrow £27,414 for a sanatorium at Nab Top Farm, Marple.

Neath.—An inquiry has been held by the Local Government Board into an application of the Glamorgan County Council for sanction to a loan of £14,000 for the provision of an isolation hospital at Cwmla, Neath.

Norwich.—Plans have been prepared by Messrs. E. Boardman and Son, of Norwich, for an emergency hospital to be built in the grounds of the Norfolk and Norwich Hospital.

### THE LONDON SCHOOL OF TROPICAL MEDICINE.

The following have passed the examination at the end of the forty-sixth session of the London School of Tropical Medicine:—

W. I. Escoffery, M.B., B.Ch. Aber. (Colonial Service), with distinction. Dr. Escoffery has been awarded the Duncan Medal of the School, which is awarded to the student who obtains the highest aggregate of marks during the session.

G. C. S. Perera, L.M.S., Ceylon (Colonial Service); A. E. Schokman, M.R.C.S., L.R.C.P.; C. Deuntzer, M.R.C.S., L.R.C.P.; E. F. Wills, M.B., C.M. Edin.; J. E. Moffatt, M.R.C.S., L.R.C.P. Ireland (Colonial Service); and E. B. Bate, M.B. Dublin (Colonial Service).

### Medical Appointments.

The following appointments have been made at St. Thomas's Hospital: Resident Assistant Surgeon, S. Rouquette, M.A., M.B., M.C. Cantab., F.R.C.S. Eng.; Medical Registrar, G. Hoffmann, B.A., M.B., B.C. Cantab., M.R.C.P. Lond.; Surgical Registrar, P. H. Mitchiner, M.B., M.S. Lond., F.R.C.S. Eng.; Obstetric Registrar, J. M. Wyatt, M.B., B.S. Lond., F.R.C.S. Eng.; Ophthalmic Registrar, G. W. Spencer, B.A., M.B., B.C. Cantab., M.R.C.S., L.R.C.P.

### Personalia.

At a recent meeting of the Edmonton Board of Guardians it was decided to send a congratulatory letter to Dr. A. W. Gregorson, their assistant medical officer, on his obtaining the degree of M.D., with commendation, of the University of Glasgow.

Dr. C. H. Browne, district medical officer for Ponders End, who has joined the Navy as temporary surgeon for the period of the war, has been officially informed that the Guardians will keep open his appointment under the Board until his return from service with the Navy.

### Obituary.

The death is announced, after a very short illness, of Dr. J. Ward Lawson, M.R.C.S., L.R.C.P. (Lond.), district medical officer for the St. Pancras Guardians, London. This is a particularly sad occurrence, as Dr. Lawson had only been married quite recently. The Guardians at a recent meeting decided to send a note conveying their sympathy to Mrs. Lawson.

### Institutional Needs.

REDUCING COAL BILLS.

As combustion engineers, Meldrums, Limited, have devised at their works at Timperley, near Manchester, a series of smokeless stokers, economical furnaces, steam-jet elevators, air-compressors, and incinerators for institutional use which in many respects have set the standard for efficiency. For reducing coal bills and preventing smoke their designs are remarkable, and the smokeless stoker—recently illustrated in The Hospital—when fitted to steam boilers is calculated to reduce the amount of coal burnt, and consequently the coal bill, by as much as one-fourth. The war will certainly have the effect of making institutional managers pay even more attention to consumption-saving devices than usual; and as the art of economy is best seen in the elimination of waste products, by finding uses for "waste" which was thought to serve no purpose before, a study of the Meldrum catalogues is particularly instructive reading at this time. Here mention can only be made of the steam-jet ejectors, and boiling-jets for heating water by means of live or exhaust steam, but it is in the Meldrum smokeless stokers and patent furnaces that institutional managers realising the value of economy in fuel bills and good results will be perhaps mainly interested at the moment.

Rates of Subscription : Twelve months, 6/6 ; Six months, 4/- ; Three months, 2/-, post free to any address in the United Kingdom. Abroad, Twelve months, 8/8 ; Six months, 5/- ; Three months, 2/6, post free.—Address The Subscription Dept., "The Hospital," The Hospital Building, 28/29 Southampton Street, Strand, London, W.C.

